# Prevalence of and risk factors for methicillin-resistant *Staphylococcus aureus* nasal carriage in the West of Iran: a population-based cross-sectional study

**DOI:** 10.1186/s12879-019-4567-1

**Published:** 2019-10-28

**Authors:** Elham Ahmadi, Mohammad Khojasteh, Seyed Mohammad Mortazavi, Fatemeh Khan-Mohammadi, Ali Kazemnia, Javad Beheshtipour, Mahdieh Raeeszadeh

**Affiliations:** 1 Department of Pathobiology, Faculty of Veterinary Medicine, Sanandaj Branch, Islamic Azad University, Sanandaj, Iran; 2 Department of Microbiology, Faculty of Basic Sciences, Sanandaj Branch, Islamic Azad University, Sanandaj, Iran; 3 Department of Microbiology, Faculty of Veterinary Medicine, Urmia University, Urmia, Iran; 4 Young Researchers and Elite Club, Sanandaj Branch, Islamic Azad University, Sanandaj, Iran; 5 Department of Basic Sciences, Faculty of Veterinary Medicine, Sanandaj Branch, Islamic Azad University, Sanandaj, Iran

**Keywords:** CA-MRSA, SCC*mec*, *Pvl*, Antibiogram, Iran

## Abstract

**Background:**

Several reports designate the recent increase in community-acquired methicillin-resistant *Staphylococcus aureus* (CA-MRSA) nasal carriage. Because of the scanty information regarding the nasal carriage sate of MRSA in the west of Iran, the purpose of the present study was to determine the frequency of CA-MRSA in Sanandaj city.

**Methods:**

Swabs collected from anterior nares of 600 volunteers were analyzed for the presence of *S. aureus*. The isolates were further investigated for methicillin resistance by using the cefoxitin disk diffusion test, followed by PCR-amplification of the *mec*A gene. SCC*mec* types and the presence of the Panton-Valentine Leukocidin (*pvl*) encoding genes were determined through PCR. Finally, the antimicrobial susceptibility of the isolates was determined by the agar diffusion method.

**Results:**

Nasal screening identified 181 *S. aureus*, of which 55 isolates were MRSA. SCC*mec* types IV and V were detected in MRSA at frequencies of 80 and 20%, respectively. The overall frequency of *pvl* genes among the MRSA isolates was 14.54%. MRSA isolates were highly susceptible (98.18%) to mupirocin, gentamicin, and fusidic acid.

**Conclusions:**

The high prevalence of CA-MRSA carriage in the population could pose a serious public health concern for the region. Additionally, advent of drug-resistant *pvl*-positive strains demands continuous surveillance on the colonization state of CA-MRSA in order to prevent dissemination of the bacterium in the community.

## Background

*Staphylococcus aureus* is one of the major human infectious agents, causing mild to life-threatening manifestations. Anterior nares is the most consistent ecological niche for *S. aureus* in humans [1]. Almost 50% of the healthy individuals harbor the bacterium in a persistent or intermittent status, asymptomatically [2]. It is documented that the endogenous source of *S. aureus* is responsible for almost 80% of staphylococcal infections in carriers [3]. In addition to pervasive increasing in *S. aureus* reservoirs, the advent of community-acquired methicillin-resistant *S. aureus* (CA-MRSA), which is generally multi or extensively drug-resistant, has complicated the therapeutic trials in recent years [4, 5]. Acquisition of *mec*A leads to encoding a low affinity penicillin-binding protein (PBP2a). Insertion of the mobile genetic elements carrying *mec*A in the bacterial chromosome nominates staphylococcal cassette chromosome *mec* (SCC*mec*) elements, among which types IV and V are carried by CA-MRSA [6, 7]. Further, distribution of diverse antimicrobial resistant genes within the community via resident strains is the other aspect of hazard imposed by CA-MRSA [8]. It is reported that traditional Hospital-acquired MRSA (HA-MRSA) are replaced by CA-MRSA [9]. Likewise, *pvl*-harboring CA-MRSA clones may involve in necrotizing pulmonary and skin infections through the expression of Panton-Valentine Leukocidin (PVL) toxin [10].

Despite the studies carried out on CA-MRSA carriage in various part of the world [11–14], and among healthy children [15, 16], medical and non-medical students [8], and outpatients [17, 18] in Iran, the prevalence of CA-MRSA in the community setting in the west of Iran is totally unknown. Therefore, this study was conducted to evaluate the nasal colonization state of MRSA on healthy individuals in Sanandaj city, west of Iran. Moreover, SCC*mec* types, presence of *pvl*, and antibiotic resistance profile of the isolates were further analyzed.

## Methods

### Samples and data collection

The frequency of *S. aureus* nasal carriage (SANC) and MRSA was investigated in a cross-sectional study from February 2017 to July 2018 among 600 randomly chosen inhabitants in Sanandaj city based on the non-probability haphazard sampling type. Any sign of clinical illness, hospitalization, and use of antibiotics during the 12 preceding months were excluded from the survey. The participants included 278 female and 322 male. The study was approved by the Ethics Committee of Sanandaj Medical University. An informed consent signed by all the participants or their legal guardians was obtained prior to enrollment. Details of the sampling population are depicted in Table 1.

**Table 1:** Age group and sex characteristics, and distribution of SANC and MRSA

Age	Female volunteers (*n* = 278)	SANC (*n* = 68)	MRSA (*n* = 13)	Male volunteers (*n* = 322)	SANC (*n* = 113)	MRSA (*n* = 42)
1–10	31	12	3	45	22	7
> 10–20	27	11	4	39	27	12
> 20–30	34	7	1	42	13	3
> 30–40	67	3	1	52	9	3
> 40–50	58	6	2	54	12	6
> 50–60	33	14	1	46	18	9
> 60–70	28	15	1	44	12	2

### *S. aureus* isolation

Sterile swabs moisture with sterile normal saline (SNS) was used for streaking both anterior nares of each participant. The swabs embedded in SNS were transferred to the microbiology laboratory within maximum two hours and streaked onto Columbia agar (CA, Merck, Germany) with 6% defibrinated sheep blood. An individual presumptive colony of *S. aureus* (round and convex with an approximate of 1–4 mm in diameter) from each plate was subcultured on blood agar (BA, Merck, Germany). Phenotypic characterization of the *S. aureus* isolates were performed based on Gram staining and the biochemical reactions including catalase, coagulase, and DNase positive bacteria, in addition to yellow appearance on mannitol salt agar (MSA, Merck, Germany).

### DNA extraction and molecular analysis

Genomic DNA of the *S. aureus* isolates were extracted using gram positive bacterial DNA extraction kit (CinnaGen, Iran). The DNA of the isolates were subjected to a species-specific polymerase chain reaction (PCR) based on the *nuc* gene partial amplification [19]. Initial determination of MRSA was performed using cefoxitin disk (Patanteb, Iran) diffusion test [20], which was followed by *mec*A gene amplification [21]. SCC*mec* types (I-V) of MRSA isolates were discriminated in a multiplex PCR reaction as described by Ghaznavi-Rad et al. [22]. Finally, the presence of *pvl* gene was analyzed as proposed by Lina et al. [23]. Reference strain of *S. aureus ATCC* 49775 was applied as *pvl* positive control. Positive controls for SCCmec PCR were as followed: NCTC10442 (SCC*mec* I), NCTC N315 (SCC*mec* II), NCTC 85/2082 (SCC*mec* III), HDE288 (SCC*mec* IV), and JCSC3624 (SCC*mec* V).

### Antimicrobial susceptibility testing

Agar disk diffusion method was exploited to assess the antimicrobial susceptibility pattern of isolated *S. aureus* strains [20]. The used antibiotic disks (Patanteb, Iran) included rifampin (5 μg), tetracyclin (30 μg), chloramphenicol (30 μg), fusidic acid (10 μg), trimethoprim-sulphamethoxazole (1.25/23.75 μg), clindamycin (2 μg), gentamicin (10 μg), erythromycin (15 μg), penicillin (10 U), vancomycin (30 μg), mupirocin (200 μg), and ciprofloxacin (5 μg). *S. aureus* ATCC 25923 was used for quality control point.

### Statistical analysis

Demographic characteristics (gender and age-group variables) and risk factors of the studied population were primarily analyzed by bivariate model. *P* values ≤0.05 indicated statistically significant correlations at the 95.0% confidence level. Next, multiple logistic regression model was applied. In order to elevate the value of regression model, every given variable with a *P* value ≤0.05 was entered in the final multiple logistic model. The goodness of fit regarding multiple logistic regression model was evaluated by Hosmer and Lemeshow test. The results were expressed as *P* value and adjusted odds ratio (Adjusted OR) with a 95% confidence interval (CI 95%). Median ages expressed as mean ± SEM.

## Results

In the present research, 181 (30.16%) colonized *S. aureus* isolates were detected, among which 68 (11.33%) and 113 (18.83%) strains were isolated from female and male subjects, respectively. The overall prevalence of MRSA in this study was 55 out of 181 *S. aureus* isolates, identified phenotypically and then confirmed in a *mec*A-based PCR assay. The most affected age-group among females and males were > 60–70 and > 10–20 years-old groups, respectively. Thirteen (2.16%) and 42 (7%) female and male candidates were detected as MRSA nasal carriers, respectively (Table 1).

The individuals with highest carriage rate of MRSA in both genders were in > 10–20 age group. The prevalence of MRSA in men was rather higher than that of women (Table 2). The median age of women and men enrollers were 36.27 ± 1.06 and 36.08 ± 1.09 years, respectively. The risk of MRSA (Adjusted OR: 3.86; 95% CI: 1.70–8.76) infection was significant with gender (Table 3). Meanwhile, significance association was observed between MRSA frequency with age (Adjusted OR: 0.92; 95% CI: 0.78–1.10) (Table 3). Besides, in bivariate logistic regression analysis, a significant association was revealed between the frequency of MRSA with some independent risk factors including skin and soft tissue infection in the last 12 months (Crude OR = 46.57, 95% CI: 2.03–1068.89), occupation (Crude OR = 1.32, 95% CI: 1.24–3.52), and the presence of household members who have a profession in hospital care (Crude OR = 13.07, 95% CI: 2.44–70.11) (Table 2). All of the above mentioned risk factors remained associated in the multiple logistic regression analysis, including skin and soft tissue infection in the last 12 months (Adjusted OR = 4.70, 95% CI: 1.22–18.18), occupation (Adjusted OR = 0.22, 95% CI: 0.09–0.51), and the presence of household members who have a profession in hospital care (Adjusted OR = 5.24, 95% CI: 1.86–14.76) (Table 3). No statistical relationship was identified between the other risk factors depicted in Table 2 and MRSA state. The *P*-value, crude odds ratio and confidence interval (CI) of the risk factors are accessible in Table 2.

**Table 2:** Bivariate analysis of demographic information and risk factors for MRSA

Factor	SANC Frequency (% column)	MRSA		*p*	Crude OR	95% CI
		Positive No. (%)	Negative No. (%)			
Sex						
Male	113 (35.09)	42 (37.17)	71 (62.83)	0.001	6.48	2.13–19.66
Female	68 (24.46)	13 (19.12)	55 (80.88)			
Age^a^						
1–10 years	34 (44.74)	10 (29.41)	24 (70.59)	–	1	–
10–20 years	38 (57.58)	16 (42.10)	22 (57.90)	0.002	10.64	2.37–47.79
20–30 years	20 (26.32)	4 (20.00)	16 (80.00)	0.78	1.33	0.17–10.52
30–40 years	12 (10.08)	4 (33.33)	8 (66.77)	0.48	2.18	0.25–19.29
40–50 years	18 (16.07)	8 (44.44)	10 (55.56)	0.05	8.85	1.02–76.89
50–60 years	32 (40.51)	10 (31.25)	22 (68.75)	0.08	4.62	0.83–25.53
60–70 years	27 (37.50)	3 (11.11)	24 (88.99)	0.19	0.12	0.004–3.03
Hospital visit in the last 3 months						
Yes	58 (32.77)	20 (34.48)	38 (65.51)	0.09	2.96	0.85–10.36
No	123 (29.08)	35 (28.45)	88 (71.54)			
Chronic renal disorder						
Yes	27 (39.71)	11 (40.74)	16 (59.25)	0.91	1.14	0.14–9.01
No	154 (28.95)	44 (28.57)	110 (71.42)			
Smoking habits^a^						
Ex-smoker	22 (44.90)	4 (18.18)	18 (81.81)	–	1	–
Current smoker	39 (31.97)	16 (41.02)	23 (58.97)	0.09	12.96	0.64–263.94
Non-smoker	120 (27.97)	35 (29.16)	85 (70.83)	0.23	6.4	0.3–136.99
Occupation						
Yes	97 (35.93)	22 (22.68)	75 (77.31)	0.04	1.32	1.24–3.52
No	84 (25.45)	33 (39.28)	51 (60.71)			
Household members who have a profession in hospital care						
Yes	36 (47.37)	16 (44.44)	20 (55.55)	0.003	13.07	2.44–70.11
No	145 (80.11)	39 (26.89)	106 (73.10)			
Skin and soft tissue infection in the last 12 months						
Yes	14 (36.84)	9 (64.28)	5 (35.71)	0.02	46.57	2.03–1068.89
No	167 (29.72)	46 (27.54)	121 (72.45)			
Allergy						
Yes	15 (22.39)	3 (20.00)	12 (80.00)	0.06	0.1	0.01–0.82
No	166 (31.14)	52 (11.32)	114 (88.68)			
Diabetes mellitus						
Yes	7 (36.84)	1 (14.28)	6 (85.71)	0.9	0.04	0.001–1.62
No	174 (29.95)	54 (31.03)	20 (68.96)			
Surgery in the last 12 months						
Yes	4 (26.67)	1 (25.00)	3 (75.00)	0.82	0.65	0.02–25.57
No	177 (30.26)	54 (30.50)	123 (69.49)			
Chronic liver disorder						
Yes	0 (0.00)	0 (0.00)	0 (0.00)	1	–	–
No	181 (30.37)	55 (30.38)	126 (69.61)			
Use of steroids						
Yes	3 (25.0)	1 (33.33)	2 (66.66)	0.22	13.95	0.21–912.44
No	178 (30.27)	54 (30.38)	124 (69.66)			
Oral contraceptive						
Yes	8 (21.62)	3 (37.5)	5 (62.5)	0.31	4.61	0.24–89.79
No	173 (30.73)	52 (30.05)	121 (69.94)			
Alcoholism						
Yes	7 (41.18)	3 (42.85)	4 (57.14)	0.388	4.04	0.176–92.58
No	174 (29.58)	52 (29.88)	122 (70.11)			

^a^ Regression was used with the first value within variable acting as reference value

Variables entered: sex (male versus female), and the other variables, yes versus no.

OR: Odds ratio

CI: Confidence interval

**Table 3:** Multiple logistic regression analysis of demographic information and risk factors associated with MRSA

Risk factors	*p*	Adjusted OR	95% CI
Sex	0.001	3.86	1.70–8.76
Age	0.038	0.92	0.78–1.10
Skin and soft tissue infection in the last 12 months	0.03	4.70	1.22–18.18
Occupation	0.001	0.22	0.09–0.51
Household members who have a profession in hospital care	0.002	5.24	1.86–14.76

OR: Odds ratio

CI: Confidence interval

A total of 44 (80%) MRSA isolates carried SCC*mec* type IV, among which eight isolates harbored *pvl* gene. The frequency of *pvl* and SCC*mec* type IV subtypes are represented in Table 4. In addition, SCC*mec* type V was detected in 11 (20%) MRSA isolates and only one of them harbored *pvl* gene. Other types of SCC*mec* including types I, II, and III were not detected in any isolates.

**Table 4:** Frequency of SCC*mec* type IV subtypes among MRSA isolates

Subtype	Female	Male	Total
IVa	3 (5.45%)	15 (27.27%)	18 (32.72%) *
IVb	0 (0%)	4 (7.27%)	4 (4.27%) *
IVc	2 (3.63%)	11 (20%)	13 (23.63%) *
IVd	1 (1.81%)	0 (0%)	1 (1.81%)
IVh	3 (5.45%)	5 (9.09%)	8 (14.54%) *
Total	9 (16.36%)	35 (63.63%)	44 (80%)

* Strains harbored *pvl* included 3 isolates in each IVa and IVc subtypes and 1 isolate in each IVb and IVh subtype

The antibiotic resistance pattern of *S. aureus* including MRSA is presented in Table 5. Drug-resistance (resistance to at least 2 classes of antibiotics) was observed in 132 (72.92%) and 47 (87.27%) total *S. aureus* and MRSA isolates, respectively. Resistance to penicillin allocated the highest rate of resistance, following by clindamycin, erythromycin, and ciprofloxacin among the total and MRSA isolates. All MRSA isolates were susceptible to fusidic acid, gentamicin, and mupirocin, except one isolate. Besides, high rates of susceptibility were observed against the three above antibiotics in total isolated *S. aureus*.

**Table 5:** Antibiotic resistance profile of *S. aureus* isolated from nasal carriers in Sanandaj city

Antibiotic	*S. aureus* *n* = 181			MRSA *n* = 55		
	R n (%)	I n (%)	S n (%)	R n (%)	I n (%)	S n (%)
Rifampin	15 (8.28%)	8 (4.41%)	158 (87.29%)	3 (5.45%)	1 (1.81%)	51 (92.72%)
Tetracyclin	33 (18.23%)	0 (0%)	148 (81.76%)	13 (23.63%)	0 (0%)	42 (76.36%)
Chloramphenicol	39 (21.54%)	6 (3.31%)	136 (75.13%)	8 (14.54%)	1 (1.81%)	46 (83.63%)
Fusidic acid	6 (3.31%)	0 (0%)	175 (96.68%)	1 (1.81%)	0 (0%)	54 (98.18%)
Trimethoprim-sulphamethoxazole	17 (9.39%)	2 (1.10%)	162 (89.50%)	4 (7.27%)	0 (0%)	51 (92.72%)
Clindamycin	142 (78.45%)	9 (4.97%)	30 (16.57%)	32 (58.18%)	4 (7.27%)	19 (34.54%)
Gentamicin	4 (2.20%)	2 (1.10%)	175 (96.68%)	1 (1.81%)	0 (0%)	54 (98.18%)
Erythromycin	92 (50.82%)	16 (8.83%)	73 (40.33%)	27 (49.09%)	2 (3.63%)	26 (47.27%)
Penicillin	173 (95.58%)	0 (0%)	8 (4.41%)	55 (100%)	0 (0%)	0 (0%)
Vancomycin	21 (11.60%)	9 (4.97%)	151 (83.42%)	6 (10.90%)	3 (3.63%)	46 (83.63%)
Mupirocin	3 (1.65%)	1 (0.55%)	177 (97.79%)	0 (0%)	1 (1.81%)	54 (98.18%)
Ciprofloxacin	87 (48.06%)	11 (6.07%)	83 (45.85%)	16 (29.09%)	3 (3.63%)	36 (65.45%)

R: resistant; I: intermediate; S: sensitive; n: number

## Discussion

Emergence and distribution of CA-MRSA clones is a universal public health issue. High frequency of colonized *S. aureus* detected in the present survey is a matter of concern as nasal carriers are at risk of acquiring endogenous Staphylococcal infections [8]. Relatively poor personal hygiene such as facial, hand, and nostril cleaning habit is a probable reason of the high rate of SANC in the region [24]. Although a reverse association has been stated between SANC and age [14], higher prevalence of SANC among elderly women in this study is ambiguous. This may be related to hormonal disposition [13]. As an evidence, consumption of hormonal contraceptives is identified as a risk factor for SANC [25]. On one hand, female sex hormones direct an immunomodulating effect which may modify the host innate and adaptive responses against infections. This is particularly about estrogen through the exertion of anti-inflammatory and pro-inflammatory effects at different physiological levels [26, 27]. On the other hand, the dynamics of SANC is indirectly affected by the existence of sex steroid receptors in the anterior nares, in addition to the microenvironment of nasal cavity which is influenced by sex hormones [24, 28]. The peak competition of respiratory pathogens to colonize in the anterior nares is in the first years of life, by which interfering of the bacteria confers their elimination or establishment [1]. Higher prevalence of SANC state is consistently documented in male gender [8, 13, 14]. Despite this, some observations have documented no differences regarding the SANC state in both genders [29]. Empirically experience of the authors denotes the less habit of hand washing among young boys. Although the impact of gender on SANC frequency is not completely known so far, enhanced attitude of female sex toward health issues, particularly personal hygiene, may influence the bacterial colonization rate [8].

The reported incidences of SANC in different studies vary, depending on the sampling sites and methods, and the methodology applied [12]. Lower frequency of SANC among apparently healthy individuals have been reported from Spain (19.1%) [12], Norway (27%) [30], Kashmir-India (27.92%) [31], and Germany (21.9%) [13], while the higher prevalence of SANC (40.4%) has been documented in Ukraine [32]. It is not out of mind that the prevalence rate of *S. aureus* in this study in under-estimated as other sites of body residing *S. aureus* (skin, vagina, pharynges) did not examined. In addition, enrichment processes did not employed for rising *S. aureus* isolation.

High level of CA-MRSA in this study poses a risk for not only carriers but also patients and susceptible individuals. The previous history of skin and soft tissue infections is independently associated with MRSA rate. Current studies have also confirmed MRSA colonization with surgical site infections or chronic furunculosis [33, 34]. Despite this, because of some controversial conflicts regarding association of MRSA colonization with several non-infectious skin diseases [35–37], the burden of CA-MRSA state in skin and soft tissue infections in the studied district is needed to investigate in details in upcoming studies. Despite the success in preventing recurrent skin infections following nasal decolonization with mupirocin [33], it has been failed in the cases of non-infectious skin diseases [38]. In other studies undertaken in Iran, the carriage state of MRSA among special groups including healthcare workers [39], children [16], students [8], and hospitalized individuals [40] were 5.3, 1.3, 13.14, and 36.8%, respectively. The nasal colonization rate of MRSA was reported 4.5% in a community based study carried out in Arak, central Iran [41]. The carriage rate of MRSA in Spain (0.4%) [12], Bolivia (0.5%) and Peru (0%) [11], Brazil (0.9%) [14], Germany (1.29%) [13], India (1.83%) [31], and Ukraine (3.7%) [32] were lower than the 9.16% detected in our study. Higher incidence of MRSA nasal carriage (16.6%) has been reported by Goud et al. [42], while Scerri et al. [43] and Onanuga et al. [44] has stated approximately close rate of MRSA nasal colonization from Malta (8.8%) and Niger delta (9%). Regular close contact with an active case or carrier, traffic to healthcare centers, poor sanitation or economic circumstances, preceding antibiotic prescription, and overcrowding are the plausible reasons to explain the differences of CA-MRSA prevalence in various regions [45, 46]. There is concordance between the results of the present work and Schaumburg et al. regarding peek colonization rate of the bacterium among teenagers [47]. The higher proportion of MRSA carriage state in male rather than female volunteers in this study complies with the results that presented elsewhere [8, 13, 30, 32]. Indeed, this is coincide with the observations reported from Arak, central Iran [48] and the USA [49].

The frequent harboring of SCC*mec* type IV among the MRSA isolates underscored their community-acquired origin. Similarly, predominate SCC*mec* type among CA-MRSA in other studies carried out in Iran [8, 41] and overseas [11, 12, 14] was type IV. IVa subtype had the most frequency in Urmia, the northwest of Iran, with the association of none detectable IVb and IVc subtypes [8]. Lozano et al. revealed IVc subtype as the dominant subtype of SCC*mec* type IV [12]. This emphasizes even the subtle genetic variations of CA-MRSA isolated in different areas.

Because of the PVL-positive CA-MRSA menace to cause soft tissue and skin infections in menage and close-contact social groups, it is assumed as a serious public health threat. The frequency of *pvl* gene among the CA-MRSA isolates in the present study was 14.54% (8 out of 55 isolates), which is higher than findings in the central [41] and the northwest of Iran [8]. While Fard-Mousavi et al. reported 20.8% prevalence for *pvl* [48]. PVL-negative CA-MRSA clones were reported from Spain [12], Bolivia [11], and Botucatu, Brazil [14]. In contrast, approximately high prevalence of *pvl*-harboring CA-MRSA isolates were detected from Ukraine (58%) [32]. Despite the prevailing distribution of *pvl* in CA-MRSA clones in Western countries rather than Eastern countries [50], mountainous subtropical climate condition of Sanandaj city may influence the higher prevalence of *pvl* in this city comparing to the mentioned European countries except Ukraine [51]. Meanwhile, due to the phage origin of *pvl* genes and their transmissibility from methicillin-sensitive *S. aureus* (MSSA) into CA-MRSA, it is highly recommended to screen MSSA for the presence of this gene [52].

Approximately 100% sensitivity to mupirocin was observed herein. This is in line with the other reports from Iran [8, 41]. Therefore, mupirocin can efficiently be used to mitigate MRSA nasal carriage [53]. Gentamicin is used as a synergic antibiotic in treatment of acute staphylococcal infections, like endocarditis. Fusidic acid is an appropriate alternative for the treatment of *S. aureus* infections in the cases of antibiotic resistance. In order to hinder the development of resistance against the above mentioned antimicrobials, diligent care in their use must be ensured. The same as the other internal data [8, 41], high rate of sensitivity against rifampin may be explained by the low prescription of this agent in medicine in Iran. Despite the reports from the northwest of Iran [8], high rate of resistance observed against clindamycin and erythromycin in this study may be a consequence of wide prescriptions of lincosamides and macrolides in treatment regime of gram positive bacterial infections in the region. Besides, there is obvious discrepancy regarding ciprofloxacin resistance rate in our study and others [8, 32, 41] which may be depend on the easy access of individuals to it without physicians prescriptions. Hence, prudent prescription and limited access of individuals to antimicrobial agents in the region is highly recommended.

## Conclusion

In conclusion, the relatively high incidence of CA-MRSA in asymptomatic individuals in the west of Iran is a worrisome matter. Hence, implementation of follow-up programs is crucial to restrict and/or interrupt transmission of the bacteria from infected carriers to in-contact persons. Presence of either drug-resistance or *pvl*-harboring isolates may emphasize more the application of further continuous surveillance in the region.

## Data Availability

The datasets generated and/or analyzed during the current study are not publicly available since the data contains particular medical records and individual privacy could be compromised, but are available from the corresponding author on reasonable request.

## Abbreviations

BA: blood agar
CA: Columbia agar
CA-MRSA: community-acquired methicillin-resistant *Staphylococcus aureus*
CI: confidence interval
HA-MRSA: Hospital-acquired methicillin-resistant *Staphylococcus aureus*
MSA: mannitol salt agar
OR: odds ratio
PBP2a: penicillin-binding protein
*pvl*: Panton-Valentine Leukocidin
SANC: *Staphylococcus aureus* nasal carriage
SCC*mec*: staphylococcal cassette chromosome *mec*
SNS: sterile normal saline
